# Two-year clinical outcomes of standalone gonioscopy-assisted transluminal trabeculotomy in normal-tension glaucoma

**DOI:** 10.3389/fmed.2026.1828249

**Published:** 2026-04-17

**Authors:** Zhengfang Wu, Suju Liu, Ping Yu, Xiao Tang, Liuzhi Zeng

**Affiliations:** 1 Chengdu University of Traditional Chinese Medicine, Chengdu, Sichuan, China; 2 Affiliated Hospital of Integrated Traditional Chinese and Western Medicine, Chengdu University of Traditional Chinese Medicine (Chengdu First People’s Hospital), High-Tech Zone, Chengdu, Sichuan, China

**Keywords:** normal-tension glaucoma, gonioscopy-assisted transluminal trabeculotomy, minimally invasive glaucoma surgery, intraocular pressure, IOP-lowering medications, safety

## Abstract

**Purpose:**

To evaluate the 2-year efficacy and safety of gonioscopy-assisted transluminal trabeculotomy (GATT) for normal-tension glaucoma (NTG).

**Methods:**

This single-center retrospective case series included 9 NTG patients (12 eyes) undergoing GATT, with follow-up at postoperative day 1, week 1, and months 1, 3, 6, 12, 18, and 24. Primary outcomes were changes in intraocular pressure (IOP) and IOP-lowering medication burden at 24 months (counted by active ingredients). Complete and qualified success were assessed using strict criteria (from postoperative month 1 onward: IOP 6–15 mmHg and ≥20% reduction from baseline). Postoperative events and additional interventions/reoperations were recorded. Paired *t* tests or Wilcoxon signed-rank tests were used as appropriate; a one-eye-per-patient sensitivity analysis was performed.

**Results:**

The median age at surgery was 65 (53–70) years, and most eyes had moderate-to-advanced glaucoma. Mean IOP decreased from 17.33 ± 3.53 mmHg to 13.33 ± 2.35 mmHg at 24 months (mean reduction 4.00 ± 2.86 mmHg; 95% CI 2.18–5.82; *t* = 4.844; *p* < 0.001), corresponding to a 23.1% reduction. Medication burden decreased from 1 (0, 2) to 0 (0, 0) (*Z* = −2.251; *p* = 0.024). At 24 months, qualified and complete success rates were both 50.0% (6/12); sensitivity analysis (*n* = 9) was consistent. No hypotony, IOP spikes, or serious complications occurred. Events were mild and self-limited (mainly hyphema and aqueous flare). No SLT, needling, or additional glaucoma surgery was required.

**Conclusion:**

GATT significantly reduced IOP and medication burden in NTG with a safety profile through 24 months. Larger prospective studies are warranted.

## Introduction

Normal-tension glaucoma (NTG) is a major subtype of open-angle glaucoma characterized by progressive optic neuropathy and visual field loss despite intraocular pressure (IOP) within the statistical “normal” range (1). Compared with high-pressure glaucoma, NTG typically has a lower baseline IOP and often requires a lower and more stable target IOP while avoiding hypotony-related complications. Although multiple mechanisms have been implicated, IOP reduction remains the most established and modifiable therapeutic target, and current guidelines emphasize individualized target IOP setting and long-term follow-up (2, 3). Medical therapy alone may be limited by adherence, local adverse effects, and long-term costs, and progression can still occur in some patients despite multidrug regimens (1). In addition, NTG is relatively common in East Asian populations, underscoring the value of real-world evidence from this region (4).

Traditional filtering surgery and tube shunt procedures can achieve low IOP levels but are associated with bleb-related complications and substantial postoperative management burden (3). Minimally invasive glaucoma surgery (MIGS) has therefore attracted attention because of its less invasive nature and generally favorable safety profile (5). A systematic review and meta-analysis in NTG suggests that MIGS can lower IOP and reduce medication burden, although heterogeneity exists due to differences in procedures, follow-up duration, and success definitions (6).

Gonioscopy-assisted transluminal trabeculotomy (GATT) is a 360° circumferential trabeculotomy that enhances physiologic aqueous outflow without creating a conjunctival filtration bleb, potentially reducing bleb-related complications and postoperative care burden (5). Multicenter cohorts and longitudinal studies in open-angle glaucoma have reported significant IOP and medication reductions with an overall favorable safety profile, and long-term studies suggest sustained IOP lowering while highlighting the need to better define reoperation risk and prognostic factors (7–9). Registry-based real-world studies further provide complementary evidence on long-term outcomes and additional interventions after angle-based MIGS (10). However, dedicated long-term data on GATT specifically in NTG remain limited, and success estimates may differ when stricter criteria aligned with NTG targets (e.g., IOP ≤ 15 mmHg and ≥20% reduction from baseline) are applied (11). Therefore, we conducted a retrospective study to evaluate 24-month outcomes of GATT alone in NTG, focusing on IOP control, changes in medication burden, strict prespecified success rates, postoperative complications, and the need for additional interventions or reoperations, to provide preliminary evidence to support the clinical application of GATT in NTG.

## Methods

### Study design and participants

This was a single-center retrospective case series. We consecutively enrolled patients with normal-tension glaucoma (NTG) who underwent gonioscopy-assisted transluminal trabeculotomy (GATT) at the Affiliated Hospital of Integrated Traditional Chinese and Western Medicine, Chengdu University of Traditional Chinese Medicine (Chengdu First People’s Hospital) between January 2020 and January 2024. A total of 9 patients (12 eyes) were included. Patient-level variables were summarized per patient, whereas eye-level outcomes were analyzed per eye.

Inclusion criteria were as follows: (1) Diagnosis of NTG: typical glaucomatous optic nerve damage with corresponding visual field defects, repeated untreated IOP measurements ≤21 mmHg, and an open anterior chamber angle on gonioscopy; (2) Age ≥ 18 years; (3) Undergoing standalone GATT (without concomitant cataract surgery or other intraocular procedures), with successful completion of a 360° trabeculotomy. (4) Complete follow-up data including at least postoperative day 1, week 1, and months 1, 3, 6, 12, 18, and 24; (5) Availability of key perioperative and follow-up outcome measures (IOP and medication use).

Exclusion criteria were: (1) Prior filtering surgery or other minimally invasive glaucoma surgery (MIGS) in the study eye; (2) Eyes with significant angle closure, secondary glaucoma, non-glaucomatous optic neuropathies, or other factors that could confound the diagnosis of NTG were excluded; (3) Concomitant cataract surgery or any other combined intraocular procedures at the time of GATT; (4) Missing follow-up data or unavailable key variables.

### Surgical technique and perioperative management

All procedures were performed by a single surgeon (Liuzhi Zeng). Two paracenteses were created at the inferotemporal and superonasal clear corneal limbus using a 15° blade, and a 1.8-mm main incision was made superotemporally. After pupillary constriction, a viscoelastic agent was injected to maintain anterior chamber stability. Under direct gonioscopic visualization (Volk Gonio Lens), a ~2-mm goniotomy was created at approximately the 7:30 o’clock position using a Zeng trabeculotome to incise the trabecular meshwork and the inner wall of Schlemm’s canal; viscoelastic was applied to the incision site to minimize bleeding. An illuminated microcatheter (iTrack 250A; Ellex iScience Inc., Fremont, CA) was then introduced into the anterior chamber through the superonasal paracentesis. The catheter tip was inserted into Schlemm’s canal and advanced counterclockwise circumferentially; viscodilation was performed intermittently during advancement (two injections per clock hour). After 360° cannulation, the catheter tip was retrieved from the opposite end of the goniotomy, and a circumferential trabeculotomy was completed by pulling the catheter to incise the trabecular meshwork and the inner wall of Schlemm’s canal. The anterior chamber was irrigated to remove viscoelastic and blood reflux, and corneal incisions were hydrated to ensure watertight closure. Tobramycin–dexamethasone ointment was applied at the end of surgery. Postoperatively, patients received topical tobramycin–dexamethasone, or prednisolone acetate plus topical antibiotics, along with diclofenac sodium and sodium hyaluronate eye drops for 2 weeks. After active anterior chamber bleeding had resolved, 2% pilocarpine eye drops were prescribed three times daily for 3 months.

### Follow-up and data collection

Patients were followed at postoperative day 1, week 1, and months 1, 3, 6, 12, 18, and 24. Data collected included: (1) patient-level characteristics (age at surgery, sex, age at diagnosis, and glaucoma stage); (2) eye-level baseline parameters (baseline IOP, highest preoperative IOP, number of IOP-lowering medications, cup-to-disc ratio, axial length, central corneal thickness, best-corrected visual acuity [logMAR], and visual field mean deviation); and (3) postoperative outcomes at each visit, including IOP and change from baseline, number of IOP-lowering medications, postoperative events/complications (e.g., hyphema, corneal edema, and anterior chamber inflammation) and their resolution, occurrence of IOP spikes or hypotony, surgical success, and any additional interventions (e.g., selective laser trabeculoplasty, needling, or additional glaucoma surgery). Visual field mean deviation and retinal nerve fiber layer thickness were assessed as exploratory outcomes when paired data were available. Medication burden was quantified by counting active ingredients: each active ingredient was counted as one medication; fixed-combination drops were counted according to the number of active ingredients (e.g., two active ingredients counted as two medications, three as three).

### Outcomes and definitions

The primary outcome was intraocular pressure (IOP) measured preoperatively and at each postoperative follow-up visit. The secondary efficacy outcome included changes in the number of IOP-lowering medications. Postoperative events included hyphema, shallow anterior chamber, corneal edema, aqueous flare, IOP spikes, and hypotony. Definition of IOP spike: Based on prior GATT/MIGS literature (12–14), an IOP spike was defined as IOP > 30 mmHg at any postoperative visit, or an increase of ≥10 mmHg compared with the preoperative baseline IOP; meeting either criterion was considered an IOP spike. Definition of hypotony: According to Levin et al. (15), numerical hypotony was defined as IOP ≤ 5 mmHg. Clinical hypotony was defined as hypotony accompanied by hypotony-related structural or functional abnormalities (e.g., choroidal detachment, shallow anterior chamber, or hypotony-related decrease in visual acuity).

### Surgical success

*Time point for success assessment*: To minimize the influence of early postoperative factors (e.g., inflammation or hyphema) on efficacy assessment, success and failure were evaluated starting from postoperative month 1. IOP values on postoperative day 1 and week 1 were used primarily to describe early safety and short-term changes. *Definitions of success*: Surgical success was categorized as complete or qualified success (11, 16). Qualified success: from postoperative month 1 onward, IOP within 6–15 mmHg and ≥20% reduction from baseline, with IOP-lowering medications allowed. Complete success: from postoperative month 1 onward, IOP within 6–15 mmHg and ≥20% reduction from baseline, without any IOP-lowering medication. The percentage IOP reduction was calculated as: IOP reduction (%) = (baseline IOP − follow-up IOP)/baseline IOP × 100. If IOP fell below 6 mmHg, transient early postoperative hypotony or measurement error was considered; failure based on IOP < 6 mmHg required persistence at two consecutive follow-up visits. Definition of failure: Failure was defined as either of the following occurring from postoperative month 1 onward (11): (i) IOP outside the prespecified range (<6 or >15 mmHg) at two consecutive follow-up visits; or (ii) the need for additional incisional glaucoma surgery (e.g., trabeculectomy, glaucoma drainage device implantation, repeat MIGS, or cyclodestructive procedures). Handling of additional interventions: Laser treatment (e.g., selective laser trabeculoplasty) and needling were recorded as additional interventions but were not counted as failure events. Definition of baseline IOP: In this study, baseline preoperative IOP was defined as the IOP recorded during routine preoperative clinical follow-up while patients were still using IOP-lowering medications, rather than an untreated baseline IOP obtained after a standardized medication washout.

### Exploratory outcomes

Visual function: Change in visual field mean deviation (MD) was assessed as an exploratory functional outcome, restricted to eyes with paired preoperative and 24-month visual field tests with complete data (*n* = 10). Structural outcome: Change in retinal nerve fiber layer (RNFL) thickness was assessed as an exploratory structural outcome, limited to eyes with paired preoperative and 24-month OCT RNFL measurements with complete data (*n* = 5). Exploratory outcomes were described and compared using paired analyses, and should be interpreted cautiously.

### Statistical analysis

Statistical analyses were performed using SPSS version 26.0 (IBM Corp., Armonk, NY, USA). Continuous variables were assessed for normality and are presented as mean ± standard deviation (SD) when approximately normally distributed, or as median (interquartile range) when non-normally distributed. Categorical variables are presented as counts (percentages). Paired comparisons between baseline and postoperative month 24 were performed using paired *t* tests for approximately normally distributed data and Wilcoxon signed-rank tests for non-normally distributed data. Results at other follow-up time points were primarily used to describe longitudinal trends in IOP and medication burden. To account for potential inter-eye correlation in patients with bilateral inclusion, a sensitivity analysis was performed by selecting one eye per patient using a reproducible random procedure (random number generation with a fixed seed). Baseline-to-24-month comparisons of IOP and medication burden were repeated in this one-eye-per-patient dataset to assess the robustness of the main findings. Because paired data for retinal nerve fiber layer (RNFL) thickness and visual field mean deviation (MD) were limited, these analyses were considered exploratory; statistical tests were chosen based on distributional assumptions, and Wilcoxon signed-rank tests were used when normality assumptions were not met. Success rates and their 95% confidence intervals were calculated using the exact binomial (Clopper–Pearson) method. All tests were two-sided, and *p* < 0.05 was considered statistically significant.

### Ethics statement

This study adhered to the tenets of the Declaration of Helsinki and was approved by the hospital’s Ethics Committee (Approval No. 2025YNYJ-013). Written informed consent was waived due to the retrospective design and the use of de-identified data.

## Results

### Baseline characteristics

A total of 9 patients (12 eyes) with NTG who underwent GATT were included. The median age at surgery was 65 (53–70) years, and the median age at diagnosis was 60 (50–69) years. Among the included eyes, 2 were classified as early-stage and 10 as advanced-stage glaucoma based on visual field mean deviation (MD). Mean baseline intraocular pressure (IOP) was 17.33 ± 3.53 mmHg, and the median number of preoperative IOP-lowering medications was 1 (0, 2). The median cup-to-disc ratio (C/D) was 0.8 (0.7, 0.9). Mean axial length was 24.01 ± 1.12 mm and mean central corneal thickness (CCT) was 539.17 ± 28.60 μm. The highest recorded preoperative IOP was 20.13 ± 3.25 mmHg. Median baseline best-corrected visual acuity was 0.55 (0.10–1.28) logMAR, and mean visual field mean deviation (MD) was −18.74 ± 9.75 dB. No eyes had a history of prior glaucoma surgery, and 33.33% of patients underwent bilateral GATT (Table 1).

**Table 1:** Baseline characteristics.

Variable	Value
Patients (eyes)	9 (12)
Age at surgery, years, median (IQR)	65 (53–70)
Male sex, *n* (%)	7 (77.78)
Glaucoma stage (early/advanced), eyes	2/10
Age at diagnosis, years, median (IQR)	60 (50–69)
Baseline IOP, mmHg, mean ± SD	17.33 ± 3.53
Preoperative IOP-lowering medications, no., median (IQR)	1 (0–2)
Cup-to-disc ratio (C/D), median (IQR)	0.8 (0.7–0.9)
Axial length (AL), mm, mean ± SD	24.01 ± 1.12
Central corneal thickness (CCT), μm, mean ± SD	539.17 ± 28.60
Highest recorded preoperative IOP, mmHg, mean ± SD	20.13 ± 3.25
Baseline BCVA (logMAR), median (IQR)	0.55 (0.10–1.28)
Baseline visual field MD, dB, mean ± SD	−18.74 ± 9.75
Prior glaucoma surgery, eyes (%)	0 (0)
Bilateral GATT, patients (%)	3 (33.33)

AL, axial length; BCVA, best-corrected visual acuity; CCT, central corneal thickness; C/D, cup-to-disc ratio; IOP, intraocular pressure; IQR, interquartile range; MD, mean deviation.

### Changes in IOP and medication burden

**Figure 1:**
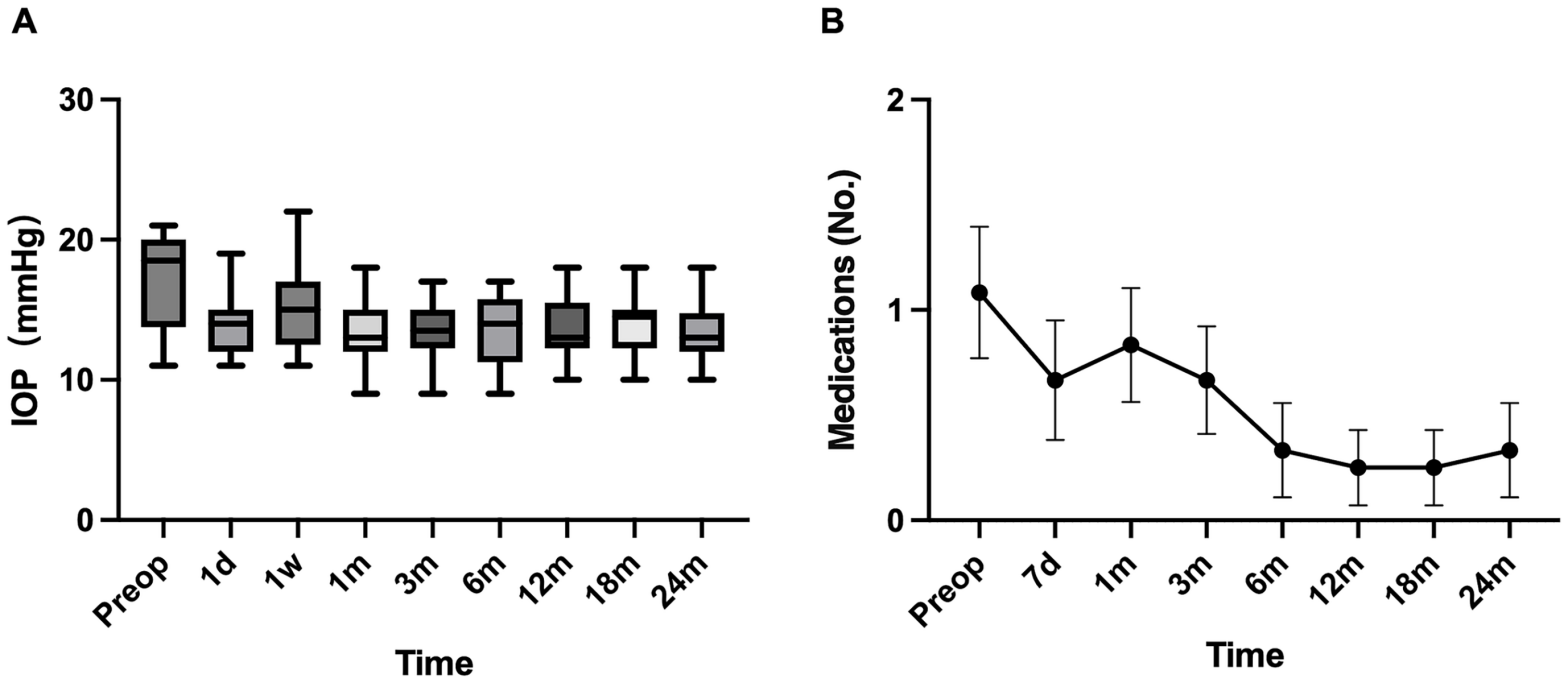
Changes in intraocular pressure (IOP) and medication burden over time after gonioscopy-assisted transluminal trabeculotomy (GATT) (*n* = 12 eyes). **(A)** Distribution of IOP across follow-up time points. Boxes represent the interquartile range (IQR) with the median shown as the horizontal line. Whiskers extend to the most extreme values within 1.5 × IQR (Tukey method). Outliers are not shown. **(B)** Mean number of IOP-lowering medications at each time point. Error bars represent the standard error of the mean (SEM). Medication burden was quantified by counting active ingredients: each active ingredient was counted as one medication; fixed-combination drops were counted according to the number of active ingredients.

Compared with baseline, IOP decreased overall at all postoperative visits and remained relatively stable throughout follow-up (Table 2; Figure 1A; mean trend shown in Supplementary Figure S1). At 24 months, mean IOP decreased significantly from 17.33 ± 3.53 mmHg to 13.33 ± 2.35 mmHg, with a mean reduction of 4.00 ± 2.86 mmHg (95% CI 2.18–5.82), corresponding to an average reduction of 23.1% (*t* = 4.844, *p* < 0.001). The number of IOP-lowering medications also decreased significantly from 1 (0, 2) at baseline to 0 (0, 0) at 24 months (*Z* = −2.251, *p* = 0.024), and remained at a low level during follow-up (Table 2; Figure 1B). In the one-eye-per-patient sensitivity analysis (*n* = 9), the direction of change was consistent with the main analysis: IOP decreased by a mean of 4.56 mmHg at 24 months (95% CI 2.34–6.77; *p* = 0.001), and medication burden also decreased (*p* = 0.038) (Supplementary Table S1).

**Table 2:** Changes in IOP and medication burden over follow-up (*n* = 12 eyes).

Time point	IOP (mmHg), mean ± SD	IOP-lowering medications, no., median (IQR)
Preoperative	17.33 ± 3.53	1 (0, 2)
Day 1	14.17 ± 2.52	–
Week 1	15.83 ± 4.11	0 (0, 2)
Month 1	13.42 ± 2.50	0.5 (0, 2)
Month 3	13.50 ± 2.36	0 (0, 1.75)
Month 6	13.50 ± 2.47	0 (0, 0)
Month 12	13.67 ± 2.27	0 (0, 0)
Month 18	13.94 ± 2.48	0 (0, 0)
Month 24	13.33 ± 2.35	0 (0, 0)
*t*/*Z* (baseline vs. month 24)	4.844	−2.251
*p* value (baseline vs. month 24)	<0.001^a^	0.024^b^

a Baseline vs month 24 IOP was compared using a paired *t* test.

b baseline vs month 24 medication burden was compared using the Wilcoxon signed-rank test.

### Surgical success

Using the prespecified success criteria (from postoperative month 1 onward: IOP 6–15 mmHg and ≥20% reduction from baseline), the qualified success rate at 24 months was 50.0% (6/12, 95% CI: 21.1–78.9%). Under the stricter definition requiring no IOP-lowering medications, the complete success rate at 24 months was also 50.0% (6/12, 95% CI: 21.1–78.9%). In an additional analysis using a stricter low-target IOP threshold, 2 of 12 eyes (16.7, 95% CI: 2.1–48.4%) achieved an IOP < 12 mmHg at 24 months; when the additional requirement of a ≥20% reduction from baseline was applied, only 1 of 12 eyes (8.3, 95% CI: 0.2–38.5%) met this criterion. No eye underwent additional glaucoma surgery, SLT, or needling during follow-up. A case-by-case summary of baseline and 24-month clinical characteristics is provided in Supplementary Table S2.

### Visual field and OCT outcomes (exploratory)

Among the 10 eyes with paired visual field examinations, mean deviation (MD) improved from −18.74 ± 9.75 dB preoperatively to −17.27 ± 9.53 dB at 24 months (*Z* = −2.191, *p* = 0.028). Paired RNFL thickness measurements were available in 5 eyes. RNFL thickness changed from 59 (49–67.5) μm at baseline to 56 (43–80) μm at 24 months, with no statistically significant difference (*Z* = −1.289, *p* = 0.197).

### Postoperative complications and additional interventions/reoperations

Postoperative events are summarized in Table 3. Hyphema occurred in 5 eyes (41.7%) and was mild in all cases, resolving spontaneously within 1 week. Aqueous flare was observed in 7 eyes (58.3%), generally mild, and gradually resolved during follow-up. Corneal edema occurred in 1 eye (8.3%) and improved with conservative management within 1 week. No eye developed numerical or clinical hypotony, IOP spikes, shallow anterior chamber, or serious complications (e.g., infection, choroidal detachment, or scleral injury). During the 24-month follow-up, no eye required additional incisional glaucoma surgery (e.g., trabeculectomy, glaucoma drainage device implantation, repeat MIGS, or cyclodestructive procedures), and no SLT or needling was performed (Table 3).

**Table 3:** Postoperative complications and additional interventions/reoperations (*n* = 12 eyes).

Event/complication	Eyes, *n*	%	Management and outcome
Hyphema	5	41.7	Mild in all cases; resolved spontaneously within 1 week; no additional intervention required
Shallow anterior chamber	0	0	None
Corneal edema	1	8.3	Conservative management (e.g., artificial tears); resolved within 1 week
Aqueous flare	7	58.3	Mild; gradually resolved during follow-up; no treatment required
IOP spike	0	0	None
Hypotony	0	0	None
Serious complications (e.g., infection, choroidal detachment, scleral injury)	0	0	None
Additional interventions/reoperations (SLT/needling/additional glaucoma surgery)	0	0	None

A single eye could experience more than one postoperative event; therefore, the total percentage may exceed 100%.

## Discussion

In this single-center retrospective case series of NTG, GATT achieved significant IOP reduction and medication sparing through 24 months, with relatively stable postoperative IOP. When assessed using the predefined strict success criteria (from postoperative month 1 onward: IOP 6–15 mmHg and a ≥20% reduction from baseline), both the qualified and complete success rates at 24 months were 50.0% (6/12, 95% CI: 21.1–78.9%). In a supplementary analysis using a stricter low-target IOP threshold, only 2 of 12 eyes (16.7%) achieved an IOP < 12 mmHg at 24 months; with the additional requirement of a ≥20% reduction from baseline, only 1 of 12 eyes (8.3%) met this criterion. Postoperative events were generally mild and self-limited, and no hypotony, IOP spikes, serious complications, or additional interventions (SLT, needling, or further glaucoma surgery) occurred during follow-up. A one-eye-per-patient sensitivity analysis yielded consistent results, supporting the robustness of the main findings.

NTG management requires achieving a lower and stable target IOP while avoiding hypotony-related complications (1–3). In this context, even modest additional IOP lowering may be clinically meaningful, particularly in moderate-to-advanced disease. The reduction in medication burden is also clinically relevant given limitations of long-term medical therapy (adherence, ocular surface adverse effects, and cost) (1). At the same time, in NTG, the ability to achieve a lower target IOP remains a key consideration in evaluating treatment efficacy. Our findings align with evidence that MIGS can lower IOP and reduce medication use in NTG, albeit with heterogeneity across procedures, follow-up duration, and outcome definitions (6, 17). Prior studies further suggest sustained IOP lowering and medication reduction after GATT in open-angle glaucoma, with transient hyphema among the most common events (18–20), and an East Asian NTG MIGS study also highlighted the clinical value of medication reduction (21).

Our success definition was stringent and tailored to NTG targets, requiring both an absolute IOP range (6–15 mmHg) and a relative reduction (≥20%) from baseline. Because baseline IOP in NTG is inherently low, achieving a ≥20% reduction is mathematically more difficult, and an upper threshold of 15 mmHg is stricter than the 18 or 21 mmHg used in many studies; therefore, success rates are not directly comparable across studies (11, 22). Particular caution is warranted because baseline IOP in this study was defined as the routine preoperative clinical IOP recorded while patients were still receiving IOP-lowering medications, rather than an untreated baseline IOP obtained after a standardized medication washout. Therefore, the estimated IOP-lowering effect and success rates based on this baseline may be conservative. In this context, the 24-month success rate of 50.0% should be interpreted cautiously, taking into account both the strict success criteria used in this study and the definition of baseline IOP. This strict dual-threshold definition may underestimate the clinical benefit in eyes that show objective improvement (e.g., IOP lowering and/or medication reduction) but do not meet both requirements. On the other hand, the stricter supplementary analysis showed a marked decline in the proportion of eyes achieving an IOP < 12 mmHg, suggesting that standalone GATT may have limited pressure-lowering capacity in NTG eyes requiring very low target IOP. Prior literature has likewise suggested that trabeculotomy and other procedures based on the trabecular meshwork/Schlemm’s canal outflow pathway may exhibit a pressure-lowering floor under stringent low-target IOP requirements (11). In contrast, filtration surgery is more likely to achieve lower IOP levels (23, 24), but at the cost of a higher risk of hypotony and a greater postoperative management burden (15). Therefore, treatment choice should still be individualized according to target IOP, safety considerations, and postoperative management burden. In other words, standalone GATT may be more suitable for selected NTG patients who wish to achieve further IOP reduction and reduce medication burden while maintaining a favorable safety profile; for patients requiring very low target IOP, whether to choose a more powerful pressure-lowering procedure should still be determined on an individual basis. Notably, no escalation of treatment occurred in our cohort, consistent with long-term studies emphasizing reintervention/reoperation as key endpoints and exploring prognostic factors (9).

Regarding safety, conventional filtering surgery or drainage devices can achieve very low IOP but carry hypotony-related risks and substantial postoperative burden (3). In contrast, angle-based procedures typically have a milder complication profile, with hyphema and transient anterior chamber inflammation being common and self-limited (7, 8, 18, 19). IOP spikes have been highlighted as early events after angle surgery/MIGS (12–14), and hypotony remains an important safety concern (15); neither was observed in our series, further supporting the overall good safety of GATT in the NTG population. Exploratory analyses were limited by incomplete paired data (MD, *n* = 10; RNFL, *n* = 5), and these findings should be interpreted cautiously. At 24 months, MD showed a mild statistical change; however, given the limited magnitude of this change, it should not be simply interpreted as definite functional improvement based solely on statistical significance, and its clinical significance remains uncertain. In contrast, RNFL thickness showed no significant change, but this finding should also be interpreted with caution because only five eyes had paired RNFL data. These results may have been influenced by the small sample size and test–retest variability; in addition, the MD findings may have been further affected by the learning effect associated with visual field testing (1). Future prospective studies with larger sample sizes and more complete paired structural and functional data are needed to more accurately evaluate the long-term effects of GATT on visual field and optic nerve structure in NTG patients.

This study is limited by its retrospective design, small sample size, and single-center setting, with potential inter-eye correlation partly mitigated by sensitivity analysis. In addition, baseline preoperative IOP in this study was derived from routine preoperative clinical measurements obtained while patients were still receiving IOP-lowering medications, as no standardized medication washout was performed before surgery. Therefore, a strictly untreated baseline IOP was not available. As a result, the percentage IOP reduction and success rates calculated from this baseline may have been influenced by ongoing medication use, and the corresponding findings should be interpreted with caution, particularly in NTG patients. From a methodological perspective, baseline IOP measured under treatment may be lower than the true untreated baseline; accordingly, the estimated pressure-lowering effect and success rates based on this baseline may be conservative. The strict success criteria may underestimate benefit in NTG with low baseline IOP; future studies should report additional, less stringent thresholds to facilitate comparisons with prior literature (11). Larger, multicenter prospective studies with longer follow-up are warranted to confirm these findings and to identify predictors of long-term success, failure, and reintervention risk in NTG (9). In conclusion, standalone GATT achieved clinically meaningful IOP reduction and medication sparing in NTG over 24 months, with a favorable safety profile; notably, no additional interventions or reoperations were required in this series, supporting its potential role as a surgical option for selected NTG patients.

## Data Availability

The de-identified datasets used and/or analyzed during the current study are available from the corresponding author on reasonable request.

## Supplementary material

FIGURE S1 Mean IOP across follow-up time points before and after GATT (*n* = 12 eyes). Data are presented as mean ± SD. The dashed horizontal line indicates 15 mmHg.
