# Supplementary figures and images for: Two-year clinical outcomes of standalone gonioscopy-assisted transluminal trabeculotomy in normal-tension glaucoma
Source: Front Med (Lausanne). 2026 Apr 17;13:1828249. doi: 10.3389/fmed.2026.1828249 (PMC13087917; doi:10.3389/fmed.2026.1828249)

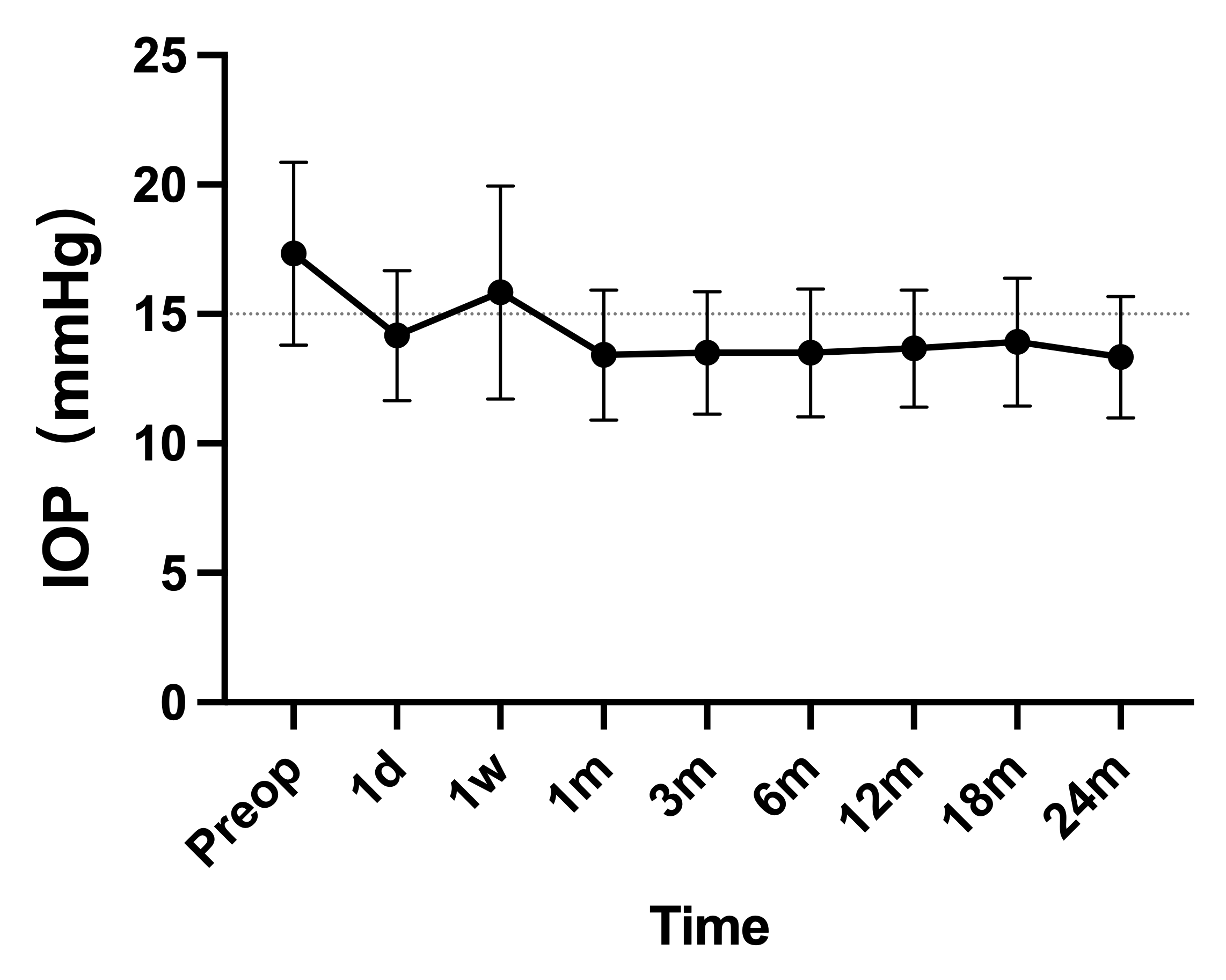

Supplement: FIGURE S1 — Mean IOP across follow-up time points before and after GATT (n = 12 eyes). Data are presented as mean ± SD. The dashed horizontal line indicates 15 mmHg. [file Image_1.TIF]
