# Supplementary material for: Two-year clinical outcomes of standalone gonioscopy-assisted transluminal trabeculotomy in normal-tension glaucoma
Source: Front Med (Lausanne). 2026 Apr 17;13:1828249. doi: 10.3389/fmed.2026.1828249 (PMC13087917; doi:10.3389/fmed.2026.1828249)
Supplement: Supplementary file 2 [file Table_1.DOCX]

**Table S1. One-eye-per-patient sensitivity analysis: changes in IOP and medication burden from baseline to 24 months (n = 9 patients)**

| **Outcome** | **Baseline** | **Month 24** | **t/Z** | **P value** |
| --- | --- | --- | --- | --- |
| **IOP (mmHg), mean ± SD** | 17.56 ± 3.54 | 13.00 ± 2.12 | 4.750 | 0.001^a^ |
| **IOP-lowering medications, no., median (IQR)** | 1（0，2） | 0（0，0） | -2.070 | 0.038^b^ |

Note: a. Baseline vs month 24 IOP was compared using a paired t test; the mean difference was 4.56 mmHg (95% CI 2.34–6.77)

b. Baseline vs month 24 medication burden was compared using the Wilcoxon signed-rank test
