# Supplementary material for: Two-year clinical outcomes of standalone gonioscopy-assisted transluminal trabeculotomy in normal-tension glaucoma
Source: Front Med (Lausanne). 2026 Apr 17;13:1828249. doi: 10.3389/fmed.2026.1828249 (PMC13087917; doi:10.3389/fmed.2026.1828249)
Supplement: Supplementary file 3 [file Table_2.DOCX]

**Table S2. Case-by-case baseline and 24-month clinical characteristics of NTG eyes undergoing standalone GATT**

| **Case** | **Age** | **Sex** | **Eye** | **Baseline IOP** | **24-month IOP** | **Baseline medications** | **24-month medications** | **VF defect type** | **24-month success status** |
| --- | --- | --- | --- | --- | --- | --- | --- | --- | --- |
| **1** | 70 | M | OD | 20 | 18 | 2 | 2 | Unavailable | 3 |
| **2** | 70 | M | OS | 19 | 17 | 3 | 2 | Unavailable | 3 |
| **3** | 54 | M | OS | 21 | 13 | 0 | 0 | Advanced diffuse loss | 1 |
| **4** | 54 | M | OD | 18 | 13 | 0 | 0 | Advanced arcuate defect | 1 |
| **5** | 51 | F | OS | 21 | 14 | 1 | 0 | Advanced superior hemifield defect | 1 |
| **6** | 51 | F | OD | 12 | 12 | 1 | 0 | Advanced superior hemifield defect | 3 |
| **7** | 65 | M | OS | 11 | 10 | 2 | 0 | Advanced arcuate defect | 3 |
| **8** | 47 | F | OD | 18 | 15 | 2 | 0 | Paracentral scotoma | 3 |
| **9** | 77 | M | OS | 16 | 11 | 0 | 0 | Advanced diffuse loss | 1 |
| **10** | 70 | M | OS | 13 | 12 | 2 | 0 | Advanced diffuse loss | 3 |
| **11** | 68 | M | OS | 20 | 12 | 0 | 0 | Advanced diffuse loss | 1 |
| **12** | 60 | M | OS | 19 | 13 | 0 | 0 | Paracentral scotoma | 1 |

Note: (1) Abbreviations: OD, right eye; OS, left eye; IOP, intraocular pressure; (2) 24-month success status: 1 = complete success; 2 = qualified success; 3 = failure; (3) Missing data are indicated as “Unavailable”.
